# Path Learning From Navigation in Aging: The Role of Cognitive Functioning and Wayfinding Inclinations

**DOI:** 10.3389/fnhum.2020.00008

**Published:** 2020-01-28

**Authors:** Veronica Muffato, Rossana De Beni

**Affiliations:** Department of General Psychology, University of Padua, Padua, Italy

**Keywords:** navigation, route learning, cognitive functioning, MoCA, pleasure in exploring, older adults, aging

## Abstract

Aging coincides with a decline in navigation and wayfinding abilities, but it is unclear to what extent factors relating to a given individual may contribute to mitigating this decline. The present study aims to analyze how older adults’ objective cognitive functioning and self-reported subjective wayfinding inclinations predict their navigation performance. Sixty-four older adults were assessed on their general cognitive functioning (all scoring from 22 to 30 on the Montreal Cognitive Assessment, MoCA), visuospatial working memory (VSWM), and perspective-taking abilities. Their self-assessed wayfinding inclinations (such as their sense of direction, pleasure in exploring places, and spatial anxiety) were also examined. Then participants learned a path in an environment from video navigation and performed a route repetition task (which maintained the same egocentric perspective as the learning phase), and a sketch map task (which involved switching from an egocentric perspective used in the learning phase to an allocentric perspective). The results showed that positive wayfinding inclinations (in terms of pleasure in exploring) related to participants’ route repetition accuracy, while their general cognitive performance (MoCA scores) related to their sketch map drawing accuracy. Individual factors such as cognitive functioning and wayfinding inclinations relate differently to older people’s navigation performance, depending on the demands of the tasks used to test their environment learning.

## Introduction

Being able to navigate in the environment is fundamental to everyday life, and any impairments in this domain can limit people’s independence and safety. Studying populations that have difficulty learning new paths in the environment, such as older adults, is consequently of particular interest. Research on pathological aging has shown that navigation deficits develop in the earliest stages of Alzheimer’s disease (AD), and even in cases of Mild Cognitive Impairment (MCI, Laczó et al., 2011). The navigation issues seen in pathological aging have inspired studies on route learning abilities in normal aging too, in an effort to monitor impairment progression, and identify the first signs of pathological aging (Lithfous et al., 2013).

After we have learned an environment from navigation, several factors can relate to the quality of our mental representation of it, or cognitive map (Tolman, 1948; Wolbers and Hegarty, 2010). Some factors are external to the individual, including the type of task used to assess this learning (Muffato et al., 2019a). Some tasks are based on egocentric knowledge of the environment and involve repeating a previously-taken path (e.g., recalling a series of turns, as in route repetition tasks). Others are based on allocentric knowledge and may involve drawing a map of the environment from memory, or recalling the location of landmarks in an area (as in sketch map tasks). Some studies found that aging affects people’s ability to repeat previously-learned routes (e.g., Barrash, 1994; Wiener et al., 2012; Taillade et al., 2016), but others identified no such impairments (e.g., Cushman et al., 2008). As for map drawing, all studies found impairments in older adults (e.g., Cushman et al., 2008; Taillade et al., 2016; Muffato et al., 2019a). These findings suggest that switching from an egocentric point of view (learning from navigation) to an allocentric one (as in map drawing tasks) is particularly problematic, even in normal aging (Lester et al., 2017). In fact, the egocentric component is better preserved than the allocentric one over an individual’s lifespan (Ruggiero et al., 2016).

Other individual factors apart from age, including cognitive abilities, and wayfinding inclinations and strategies, can relate to the features of mental representations of a path learned from navigation (Kraemer et al., 2017). In terms of cognitive abilities, studies on navigation in aging have rarely considered the role of an individual’s general level of cognition on spatial performance. The most often used measure of general cognition is the Montreal Cognitive Assessment (MoCA), a 30-item screening tool very sensitive to early changes in cognition (Nasreddine et al., 2005). It has been used as an inclusion criterion in various studies on navigation in aging (e.g., Wiener et al., 2012; Bates and Wolbers, 2014; Muffato et al., 2019a,b), but none of these studies related general cognitive performance with spatial recall performance. Only O’Malley et al. (2018) considered MoCA scores as a factor related to spatial navigation performance. They grouped older adults by their higher (26–30) or lower (22–25) MoCA scores and analyzed the two groups’ spatial performance after learning a path from navigation. The results showed a superiority of the group with higher MoCA scores in tasks that involved managing spatial information (i.e., recalling directions of landmarks), and required a change of perspective from the learning to the testing phase (i.e., choosing which of three maps depicted the route traveled in the learning phase). No differences emerged between the two groups, however, in a task resembling the format of the learning phase (i.e., recalling the sequence of landmarks). This study provided a first indication that general cognitive level may relate to spatial performance, depending on the type of spatial request. Among the various cognitive abilities, research has focused mainly on visuospatial working memory (VSWM), which retains and processes visuospatial information (Logie, 1995), and perspective-taking, which is a higher-order ability to adopt different views (Hegarty and Waller, 2004). Both are needed for navigation over the adult lifespan and are liable to age-related decline (Muffato et al., 2019b). Self-reported wayfinding inclinations—e.g., an individual’s self-rated sense of direction, pleasure in exploring places, and spatial anxiety (finding spatial demands worrying), which are not susceptible to change over time—have also been found positively related to environmental learning across the lifespan (e.g., Meneghetti et al., 2014). They may be important to good navigation performance, even in aging.

To sum up, route learning from navigation is essential for everyone and particularly important in aging. In old age, we become susceptible to a decline in our ability to manage certain types of environment knowledge, when we need to switch the perspective from the learning to the recall phase, for instance. General cognitive functioning (rarely considered in relation to navigation performance), visuospatial abilities, and self-assessed wayfinding inclinations are all factors that contribute to our navigation performance in aging, but studies conducted to date have not analyzed all these factors together.

The present study thus aims to analyze the influence of individual factors—both cognitive skills (i.e., general cognitive level and visuospatial abilities) and self-reported wayfinding inclinations—on route learning from navigation in aging. This learning is measured in terms of the ability to repeat a previously-learned path and to place the landmarks learned on a sketch map. The former is a task retaining much the same format as the learning phase, while the latter requires a change of perspective between the learning and testing phases. This is done with the novel intent to identify factors related to different environmental demands and a view to understanding the first signs of a declining ability to switch perspective that may indicate the increased risk of atypical aging. This will shed more light on the issue of age-related changes in navigation abilities.

Cognitive abilities can be expected to have a role in the task requiring a switch from egocentric to allocentric knowledge (the sketch map task). This aspect is newly explored, however, considering not only VSWM and perspective-taking ability (found to influence navigation, Muffato et al., 2019b), but also general cognitive level. The latter is measured on a continuum from low to high, considering MoCA scores from 22 to 30 (and using the cutoffs adopted by O’Malley et al., 2018) as predictors of navigation performance after accounting for the role of age from young-old to old age. After taking cognitive abilities into account, self-reported wayfinding inclinations are also expected to influence navigation performance (Muffato et al., 2019a), and are explored in relation to the demands of the task.

## Materials and Methods

### Participants

The study involved 64 healthy adults from 60 to 84 years old (34 females; *M* age = 70.55, *SD* = 7.04) who volunteered to take part and were recruited at recreation centers. A MoCA score of at least 22 (Nasreddine et al., 2005; *M* = 26.53, *SD* = 1.96) was needed in order to include only typically-aging individuals (see Bosco et al., 2017, for the Italian normative sample). Participants had attended school from 8 to 13 years old (*M* = 10.42, *SD* = 2.20), as is typical in Italy for this cohort (see ISTAT, 2011). None of the participants had a history of psychiatric, neurological or other diseases capable of causing cognitive, visual, auditory or motor impairments (Crook et al., 1986). None of them had ever visited the environment used in the learning phase.

The local ethical committee approved the study, and all participants were informed about its purposes and gave their written informed consent in accordance with the Declaration of Helsinki (World Medical Association, 2013).

### Session 1: Individual Measures

#### Objective Cognitive Measures

##### MoCA (Nasreddine et al., 2005)

This assesses multiple aspects of executive functioning, attention, working memory, delayed memory, and language. Orientation in time and place is also tested (max score 30).

##### Jigsaw Puzzle Test (JPT, De Beni et al., 2008)

This VSWM task involves mentally recomposing puzzles of objects (from 2 to 10 pieces, i.e., levels of difficulty). Participants must solve at least two of the three puzzles on a given level in order to proceed to the next. The final score is the sum of the difficulty levels of the last three puzzles solved (max 29).

##### Short Object Perspective Test (sOPT, Hegarty and Waller, 2004 and De Beni et al., 2014)

Participants have to imagine standing at one object in a layout comprising seven objects, facing another, and pointing towards a third. They indicate the direction by drawing an arrow from the center of a circle to its perimeter (six items; time limit 5 min; score 0–180° mean degrees of error; Cronbach’s α= 0.72 in the current sample).

#### Self-report Measures (From De Beni et al., 2014)

The subjective measures of participants’ wayfinding inclinations included: the *Sense of Direction and Spatial Representation scale* (SDSR; 13 items; max score 65; Cronbach’s α= 0.85), which measures an individual’s self-assessed sense of direction; the *Attitudes Towards Orientation Tasks Scale* (AtOT; 10 items; max 60; Cronbach’s *α* = 0.77) assessing their pleasure in exploring places; and the *Spatial Anxiety Scale* (SA; eight items; max 48; Cronbach’s *α* = 0.87).

### Session 2: Environment Learning and Recall Measures

#### Path Learning

A 6-min video (for details, see Muffato et al., 2019a,b) showing a walk around a botanical garden (in Padova, Italy), with 15 landmarks seen from a walker’s perspective, was projected on a screen using a.mp4 file.

#### Route Repetition Task

**Figure 1:**
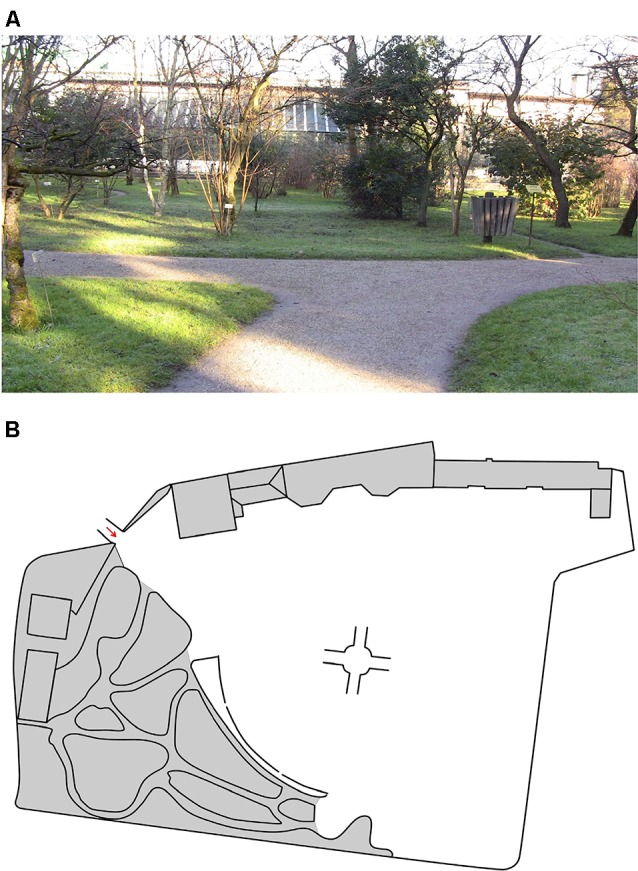
Example of the route repetition task **(A)**, and the sketch used in the sketch map task **(B)**.

This involved watching the same video again, and when it was paused, deciding which way to go along the path (with eight decision-making points; see Figure 1). If the wrong way was chosen, the feedback was provided, and the video continued in the right direction (scores: 0–8).

#### Sketch Map Task

This involved placing as many of the landmarks as possible in their right relationship to one another on a sketch map (A4 format; see Figure 1). The square root of the canonical organization was considered as a global index of accuracy (scores: 0–1; Gardony et al., 2016).

### Procedure

At a first session (lasting 45 min), participants completed a socio-demographic questionnaire, the MoCA, jigsaw puzzle test (JPT) and short object perspective test (sOPT), and the SDSR, AtOT and SA questionnaires in a balanced order. During a second session (lasting 30 min), they learned the route from the video, then performed the route repetition and sketch map tasks, in a balanced order.

## Results

The data analysis was conducted with the R software. First, mean and standard deviations of the variables and their correlations were calculated (see Table 1). Route repetition accuracy correlated directly with JPT and AtOT scores, and inversely with SA scores. Sketch map drawing accuracy correlated inversely with increasing age, directly with MoCA and JPT scores, and inversely with SA scores.

**Table 1:** Means and standard deviations of the variables and their correlations.

	*M (DS)*	1	2	3	4	5	6	7	8
1. Age	70.55 (7.04)	-
2. MoCA score	26.53 (1.96)	−0.12	-
3. JPT (VSWM)	14.11 (5.51)	**−0.40**	**0.50**	-
4. sOPT	76.40 (37.11)	**0.27**	−0.13	**−0.50**	-
5. SDSR	37.11 (8.29)	0.01	0.03	0.24	−0.12	-
6. AtOT	30.55 (8.23)	−0.15	**0.26**	**0.44**	−0.19	**0.61**	-
7. SA	22.50 (7.34)	0.24	**−0.38**	**−0.30**	0.22	−0.19	**−0.52**	-
8. Route repetition accuracy	4.78 (1.92)	−0.15	0.13	**0.26**	−0.16	0.21	**0.45**	**−0.36**	-
9. Sketch map accuracy	0.40 (0.17)	**−0.35**	**0.42**	**0.34**	−0.23	0.18	0.21	**−0.36**	**0.28**

*Note. *N* = 64; significant correlations in bold type: for |*r*| ≥ 0.25, *p* < 0.05; for |*r*| ≥ 0.33, *p* < 0.01, and for |*r*| ≥ 0.42, *p* < 0.001*.

To shed more light on the effect of the various individual factors, several linear models on route repetition and on sketch map accuracy were run stepwise to see whether the factors added at each step improved the model (changes in R^2^ are reported). Age, gender and education were entered in a baseline model (step 0) so as to examine the other factors related to spatial performance after accounting for their role. The objective factors, i.e., the MoCA as a measure of general cognitive functioning, and the JPT and sOPT (measuring VSWM and perspective-taking ability, respectively), were input in a subsequent model (step 1). Then self-reported wayfinding inclinations (SDSR, AtOT, and SA) were added in a further model (step 2) to see whether they still have a role after accounting for all the other factors investigated.

In a preliminary step, MoCA scores were considered as a dichotomous variable, high (26–30) vs. low (22–25; as in O’Malley et al., 2018), and the results were the same as when the MoCA scores were considered on a continuum. We, therefore, opted to report the latter, given the normal distribution of the MoCA scores.

For all models, the variance inflation factors revealed no significant multicollinearity (VIF values ≤ 2.57).

For the route repetition task: step 0 accounted for 11%, and step 1 for another 2% of the variance, but no predictors were significant in these steps; step 2 accounted for 13% of the variance, with pleasure in exploring places (AtOT; *β* = 0.43, *p* = 0.027) emerging as a significant predictor. As for the sketch map task: step 0 accounted for 17% of the variance, but no predictors were significant; step 1 accounted for another 14%, with MoCA score a significant predictor (*β* = 0.39, *p* = 0.005); step 2 accounted for 4% of the variance, with no factors emerging as significant predictors (see Supplementary Table S1 for estimates and *p*-values for all steps in both tasks).

## Discussion

The present study aimed to analyze the role of older adults’ objective cognitive functioning and subjective (self-reported) wayfinding inclinations on their navigation performance. The general cognitive level of a group of older adults with a broad range of MoCA scores (from 22 to 30) was here newly considered as a potential predictor of their spatial learning from navigation, alongside other cognitive aspects, such as their VSWM and perspective-taking abilities, and their self-reported wayfinding inclinations.

Our results newly showed a different involvement of certain individual factors depending on the type of recall task considered. Objective cognitive factors—and MoCA scores in particular—related to the sketch map task, which demands a switch from egocentric to allocentric knowledge. Subjective wayfinding inclinations—and especially pleasure in exploring places—related to the route repetition task, in which the perspective remains the same as in the learning phase. These results show that cognitive functioning in aging may contribute to better performance in more demanding spatial tasks after learning from navigation (O’Malley et al., 2018). This points to the importance of preserving our cognitive abilities in aging—with the aid of training programs, for instance (Lövdén et al., 2012). Moreover, detecting the very first signs of a decline in the ability to switch perspective could, therefore, be an important indicator of the risk of cognitive impairment and atypical aging (Lester et al., 2017). This issue deserves to be further investigated, administering a comprehensive neuropsychological battery to ensure the exclusion of cases of MCI. This is something not done in the present study, so our participants with lower MoCA scores may have had MCI, which could have affected their performance. Future studies should also disentangle whether the aging effect is triggered by perspective switching *per se*, or due to a generally impaired allocentric knowledge. This could be done by analyzing learning from an allocentric perspective, for instance. The other visuospatial cognitive factors considered in this study seem to not contribute to better learning from navigation, although VSWM correlated with accuracy in both tasks. On the other hand, it is worth emphasizing how self-assessed wayfinding inclinations contributed to environment learning from the navigation. After controlling for individual cognitive abilities, older adults who reported taking pleasure in exploring places were better able to solve the route repetition task. In other words, a positive attitude is helpful when addressing wayfinding tasks after learning from navigation, and an indication of a good spatial profile (Muffato et al., 2019a). In fact, pleasure in exploring correlated positively with a sense of direction and negatively with spatial anxiety, while greater spatial anxiety coincided with a weaker ability to solve spatial tasks. Promoting positive wayfinding inclinations in older people could be another way to help them maintain adequate spatial performance, for less cognitively demanding tasks at least. Finally, it is worth noting that most of the variance in the models remained unexplained. Future studies should analyze the contribution of other factors involved in navigation performance, given the great inter- and intra-individual variability in aging.

In conclusion, these results shed more light on the strengths and weaknesses of old people’s navigation abilities. External factors (such as the type of recall task) and internal factors (general cognitive abilities and self-reported wayfinding inclinations) relate differently to the features of old adults’ mental representations of environments, depending on the demands of a spatial task.

## Data Availability Statement

The data will be made available by the authors to any qualified researcher.

## Ethics Statement

The studies involving human participants were reviewed and approved by the Ethical Committee for Psychological Research at the University of Padova. The patients/participants provided their written informed consent to participate in this study.

## Author Contributions

VM and RB contributed to the design and implementation of the research, to the analysis of the results and to the writing of the manuscript.

## Conflict of Interest

The authors declare that the research was conducted in the absence of any commercial or financial relationships that could be construed as a potential conflict of interest.
