# Supplementary material for: Path Learning From Navigation in Aging: The Role of Cognitive Functioning and Wayfinding Inclinations
Source: Front Hum Neurosci. 2020 Jan 28;14:8. doi: 10.3389/fnhum.2020.00008 (PMC6997341; doi:10.3389/fnhum.2020.00008)
Supplement: Supplementary file 1 [file Table_1.docx]

Supplementary Material

# Supplementary Table 1*.* Regression models with R^2^, estimates, standardized estimates (β) and *p* values for each predictor of route repetition and sketch map accuracy.

|  |  | | **Route repetition task** | |  | | **Sketch map task** | |
| --- | --- | --- | --- | --- | --- | --- | --- | --- |
|  | ΔR^2^ | *Estimates* | β | *p* | ΔR^2^ | *Estimates* | β | *p* |
| *Step 0 (baseline)* | .11 |  |  |  | .17 |  |  |  |
| Age |  | -0.01 | -0.03 | 0.820 |  | -0.01 | -0.26 | 0.053 |
| Gender |  | 0.73 | 0.19 | 0.125 |  | 0.00 | 0.01 | 0.964 |
| Schooling years |  | 0.22 | 0.25 | 0.069 |  | 0.02 | 0.24 | 0.075 |
| *Step 1 (objective factors)* | .02 |  |  |  | .14 |  |  |  |
| MoCA scores |  | 0.03 | 0.03 | 0.857 |  | **0.03** | **0.39** | **0.005** |
| JPT (VSWM) |  | 0.05 | 0.13 | 0.466 |  | 0.01 | 0.06 | 0.701 |
| sOPT |  | 0.00 | 0.01 | 0.926 |  | -0.01 | -0.10 | 0.451 |
| *Step 2 (subjective factors)* | .13 |  |  |  | .04 |  |  |  |
| SDSR |  | -0.03 | -0.11 | 0.486 |  | 0.01 | 0.21 | 0.168 |
| AtOT (pleasure in exploring) |  | **0.10** | **0.43** | **0.027** |  | 0.01 | 0.11 | 0.529 |
| SA |  | -0.03 | -0.13 | 0.405 |  | -0.01 | -0.16 | 0.276 |
| Total R^2^ | .26 |  |  |  | .35 |  |  |  |

*Note*. *N* = 64; JPT= Jigsaw Puzzle Test; sOPT = short Object Perspective Test; SDSR = Sense of Direction and Spatial Representation scale; AtOT = Attitudes to Orientation Tasks; SA = Spatial Anxiety; significant predictors in bold type.
